# Antimicrobial Effects of Violacein against Planktonic Cells and Biofilms of Staphylococcus aureus

**DOI:** 10.3390/molecules22101534

**Published:** 2017-09-25

**Authors:** Andressa H. M. Batista, Anne C. D. Moreira, Rafael M. de Carvalho, Gleilton W. P. Sales, Patrícia C. N. Nogueira, Thalles B. Grangeiro, Suelen C. Medeiros, Edilberto R. Silveira, Nádia A. P. Nogueira

**Affiliations:** 1 Clinical Department of Toxicological Analysis, Faculty of Pharmacy, Federal University of Ceará, Fortaleza 60356000, Brazil; annecdm@gmail.com (A.C.D.M.); rafa_mendes@msn.com (R.M.d.C.); gleilton@hotmail.com (G.W.P.S.); accioly@gmail.com.br (N.A.P.N.); 2 Organic Chemistry Department, Federal University of Ceará, Fortaleza 60356000, Brazil; patufc2006@yahoo.com.br (P.C.N.N.); edil@ufc.br (E.R.S.); 3 Biology Department, Federal University of Ceará, Fortaleza 60356000, Brazil; tbgrangeiro@gmail.com (T.B.G.); sumedeiros86@gmail.com (S.C.M.)

**Keywords:** violacein, antimicrobial activity, biofilms, *Staphylococcus aureus*

## Abstract

Violacein is an indole compound, produced by *Chromobacterium violaceum*, a bacteria present in tropical and subtropical areas. Among its numerous biological activities, its antimicrobial potential stands out. This study aims to determine the antimicrobial activity of VIO on *S. aureus* in planktonic culture and biofilms. VIO showed excellent antimicrobial activity in inhibiting and killing *S. aureus* in planktonic cultures and biofilm formation. The minimum bactericidal concentration (5 μg/mL) of VIO caused the death of *S. aureus* after 3–4 h of exposure and the minimum inhibitory concentration (1.25 μg/mL) of VIO inhibited bacterial growth within the first 8 h of contact. Biofilm formation was also strongly inhibited by VIO (1.25 μg/mL), in contrast to the higher resistance verified for *S. aureus* in mature biofilm (40 μg/mL). The high bacterial metabolic activity favored VIO activity; however, the good activity observed during phases of reduced metabolism indicates that VIO action involves more than one mechanism. Thus, VIO is a promising molecule for the development of an antimicrobial drug for the eradication of *S. aureus* infections.

## 1. Introduction

*Staphylococcus aureus*, one of the main etiological agents of acquired infections in the community and the environment [1], has a remarkable ability to adapt and an enormous ability to rapidly develop resistance to countless antibiotics [2]. Its ability to produce numerous virulence factors contributes to its pathogenicity and ability to cause a variety of infectious diseases [3].

Biofilms are heterogeneous bacterial communities, irreversibly bound to a complex matrix consisting of DNA, proteins, and polysaccharides [3], which have an altered phenotype regarding growth rate and gene transcription [4]. Microbial growth in biofilm plays an important role during infection by providing different defense mechanisms to the microorganism. The biofilm matrix can prevent the access of immune cells, such as macrophages [5], as well as promote increased tolerance of microorganisms to antimicrobial agents [6].

The ability of *S. aureus* to form biofilms on medical devices, such as catheters and prostheses, increases its virulence and contributes to treatment failure [4,7]. Compared to its planktonic state, *S. aureus* in biofilms shows a significant difference in gene expression and in its physiology [8].

Thus, due to the difficulty for antimicrobial agents to promote inhibition and eradication of *S. aureus* biofilm, the search for new therapies is extremely important for the successful treatment of infectious diseases.

**Figure 1:**
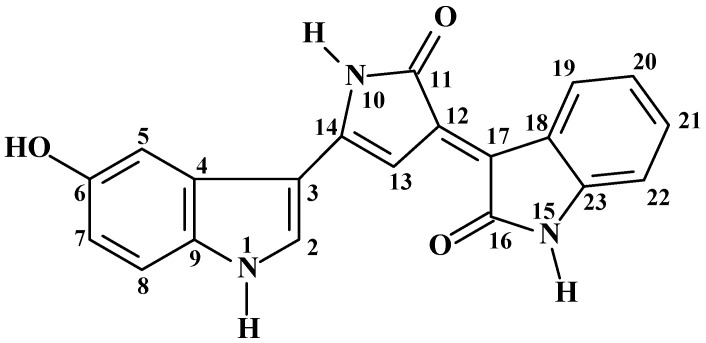
Structure of violacein from the NMR.

Violacein (VIO) is a purple bisindole metabolite, natural violet pigment produced by several Gram-negative bacteria, including the species *Chromobacterium violaceum*. VIO is a bioactive secondary metabolite, formed by the condensation of two tryptophan molecules through the action of proteins [9,10] (Figure 1).

VIO has attracted much attention in the scientific community due to its several pharmacological properties, such as antitumor, antibacterial, antiviral, antiparasitic, antioxidant action, as well as fungicide and leishmanicidal properties [10,11,12,13].

An excellent antibacterial activity of VIO has been demonstrated in vitro assays [13], as well as its synergistic potential in association with gentamicin, cefadroxil, azithromycin, and kanamycin, suggesting the possibility of using it as an alternative therapy [14], when administered in combination with other antibiotics, VIO is more effective than the use of antibiotics alone [10]. Nanoparticles loaded with VIO were two to five times more effective than VIO alone against strains of methicillin-resistant *S. aureus* (MRSA) [15].

Given the concern about the emergence of antibiotic-resistant strains, there is a great need for searching and investigating new molecules, as well as understanding their mechanism of action. In this context, VIO with its excellent antimicrobial activity especially on *S. aureus*, has emerged as a promising molecule. However, it is necessary to elucidate its antimicrobial mechanism of action so that VIO can become a new drug for the treatment of microbial infections [13,15]. The aim of this study was to determine the antistaphylococcal activity of VIO in planktonic cultures and biofilms.

## 2. Results

### 2.1. Structure Characterization

The ^1^H-NMR spectrum showed 13 proton signals, four singlets at δ 11.88, 10.72, 10.60, and 9.33; six doublets at δ 8.93 (*J* = 8.0 Hz, H-19), 8.06 (*J* = 3.1 Hz, 2 H), 7.55 (*J* = 2.0 Hz, H-13), 7.35 (*J* = 8.7 Hz, H-8), 7.23 (*J* = 2.0 Hz, H-5), and 6.83 (*J* = 8.0 Hz, H-22); two triplets of doublet at δ 7.20 (*J* = 8.0; 1.0 Hz, 21-H) and 6.95 (*J* = 8.0, 1.0 Hz, H-20); and, finally, a double doublet at δ 6.79 (*J* = 8.7; 2.0 Hz, H-7).

The singlets at δ 11.88 (NH-1), 10.72 (NH-10) and 10.60 (NH-15) were attributed to hydrogen atoms bonded to nitrogen atoms, while the singlet at δ 9.33 was associated to the proton of a hydroxyl, suggested as an oxygenated carbon substituent at δ 152.9. The ^13^C-NMR spectrum showed 20 carbon signals, in agreement with the deduced molecular formula, all related to sp2 carbons (Table 1).

All these data, together with the comparison with the NMR data in the literature [16], allowed the identification of the compound as (3*E*)-[3-(1,2-dihydro-5-(5-hydroxy-1*H*-indol-3-yl)-2-oxo-3*H*-pyrrol-3-ylidene)-1,3-dihydro]-2*H*-indol-2-one, known as VIO. The Supplementary Materials are available online. NMR are available as Supporting Information.

### 2.2. Antimicrobial Activity of VIO

The values of MIC and MBC ranged from 1.25 to 20 μg/mL and 5 to 40 μg/mL, respectively. Interestingly, VIO showed excellent activity inhibiting biofilm growth, with MBIC equal to MIC, indicating that its action occurs during biofilm formation, when the cells show intense metabolism (Table 2).

**Figure 2:**
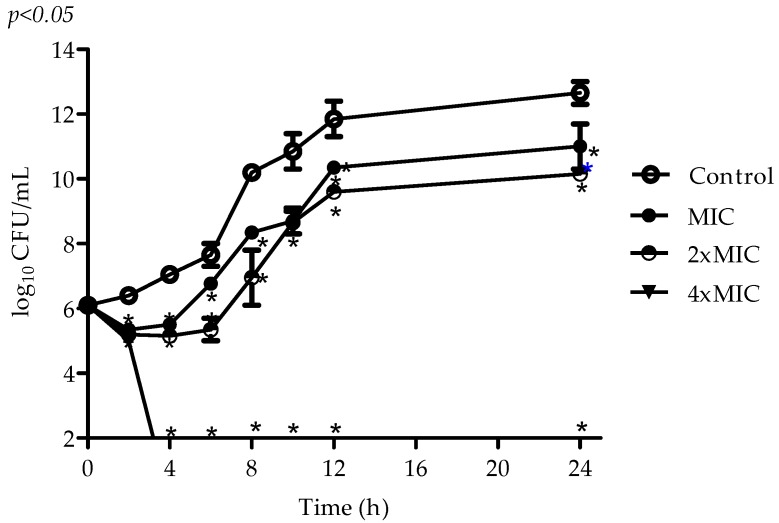
Effects of VIO on the viability of *S. aureus* ATCC 6538P. VIO (MIC = 1.25 µg/mL); Control: without VIO. Detection limit was 10^2^ colony forming units per milliliter (CFU/mL) and the assays were performed in triplicate. Data are expressed as mean ±SEM of three experiments. * *p* < 0.05 compared with the control.

The results shown in Figure 1 exhibiting the viability of planktonic cultures of *S. aureus* exposed to MIC, 2× MIC, and 4× MIC for 2, 4, 6, 8, 10, 12, and 24 h at 37 °C. VIO showed antimicrobial activity, depending on concentration, against cells of *S. aureus* ATCC 6538P, completely preventing bacterial metabolism after 3–4 h of exposure to 5 μg/mL (Figure 2).

**Figure 3:**
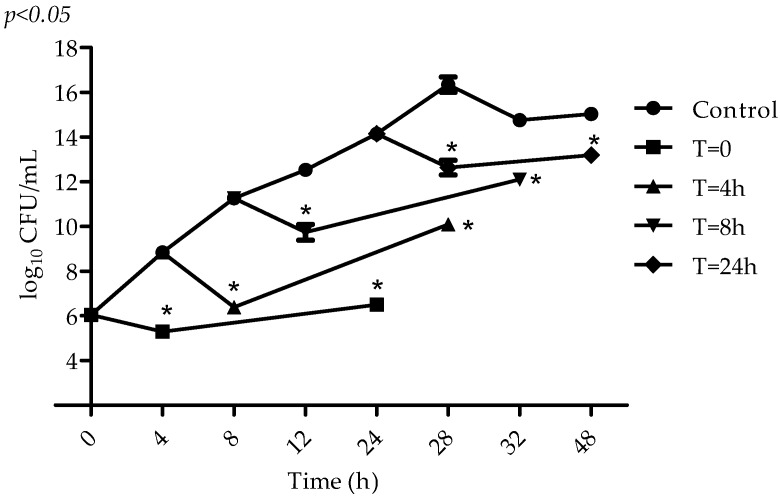
VIO effect on *S. aureus* ATCC 6538P viability at the phases of exponential and stationary growth. VIO (MIC = 1.25 µg/mL) was added at the start of incubation (*t* = 0), and after 4, 8, and 24 h. Microbial growth was assessed after 4 and 24 h of exposure. Control: without VIO. Detection limit was 10^2^ colony-forming units per milliliter (CFU/mL) and assays were performed in triplicate. * *p* < 0.05 compared with the control.

In the beginning of the exponential growth phase (4 h), when cells are young and have an intense metabolic activity, an initial reduction of 3 log_10_ CFU/mL was observed in the microbial population (Figure 3). At the end of the exponential growth phase (8 h) and stationary growth phase, VIO inhibitory action can also be observed, but to a lower intensity (1–2 log_10_ CFU/mL). 

### 2.3. Antibiofilm Activity

**Figure 4:**
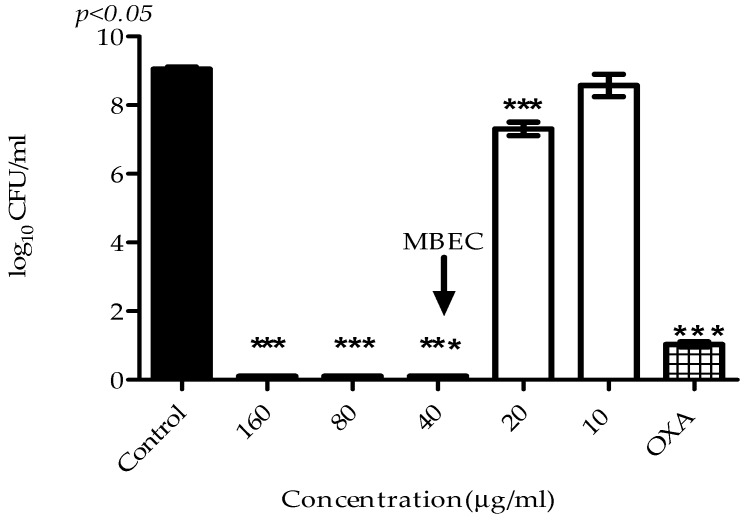
Effect of different concentrations of VIO on the viability of biofilm formed by *S. aureus* ATCC 6538P following a 24 h exposure. MBEC: minimum biofilm eradication concentration; Control: without VIO; OXA: oxacillin 20 μg/mL. Detection limit was 10^2^ colony forming units per milliliter (CFU/mL) and the assays were performed in triplicate. Data are expressed as mean ± SEM of three experiments. *** *p* < 0.05 compared with the control.

VIO effect on mature biofilm viability was the concentration of 40 μg/mL. Our results show total inhibition of bacterial metabolism and loss of *S. aureus* cell viability in mature biofilm after 150 min of exposure to MBEC (Figure 4).

**Figure 5:**
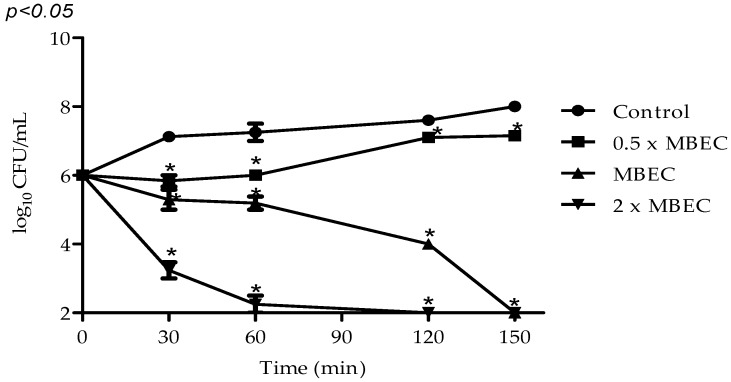
Time–kill curves for *Staphylococcus aureus* ATCC 6538P biofilm after treatment with VIO. MBEC: minimum biofilm eradication concentration (40 µg/mL); Control: without VIO. Detection limit was 10^2^ colony-forming units per milliliter (CFU/mL) and assays were performed in triplicate. Data are expressed as mean ± SEM of three experiments. * *p* < 0.05 compared with the control.

A better effect was obtained when VIO concentration was 2× MBEC, with a significantly greater reduction of cell viability (3 log_10_ CFU/mL) being observed when compared to the MBEC activity (<1 log_10_ CFU/mL) after 30 min of exposure and total cell inactivity between 90 and 120 min. The VIO subinhibitory concentration (0.5× MBEC) inhibited cell growth in biofilm when compared to the control (*p* < 0.05), but was not able to reduce the number of viable cells (Figure 5).

## 3. Discussion

Many biological activities have been identified for VIO and among them we can highlight the anti-inflammatory, anti-diarrheal, anti-ulcer, anti-tumor, antimalarial, antimicrobial, and antimycobacterial activities [10,11,13,17,18,19].

Our results show VIO antimicrobial activity on *S. aureus* strains, both resistant and susceptible to oxacillin, in suspension and in biofilm. All tested strains were susceptible to VIO and the OSSA strains showed greater susceptibility than ORSA strains.

*S. aureus* strains are often responsible for hospital infections, increasing the costs to the health system and increasing mortality and morbidity rates. Furthermore, the resistance of *S. aureus* to antimicrobial agents used in the treatment of infectious diseases hinders or even prevents treatment success, which is enhanced by its biofilm growth [20].

In order to consider VIO a molecule with potential for the development of a new chemotherapy drug for the treatment of *staphylococcal* infections, it is important to establish the kinetics of its antimicrobial action on cells in planktonic cultures and in biofilm. For that purpose, we performed the time–kill assay, which determines the time of action of a compound antimicrobial activity. The used strain was *S*. *aureus* ATCC 6538P, the most sensitive among the tested strains and the only one that had its growth in mature biofilm eradicated by the action of VIO at the tested concentration range (MBEC = 40 µg/mL). The *S. aureus* ATCC 6538P strain was also used in other assays of this study.

The VIO MIC (1.25 μg/mL) was able to inhibit the growth of *S. aureus* for up to 8 h, a period of intense metabolic activity. After this period, exponential growth was observed until 24 h of incubation, when growth was once again inhibited. The recovery of microbial growth after 8 h may be related to possible decomposition of VIO, as well as the reduction in cell activity levels, suggesting that VIO mechanism of action involves macromolecule synthesis.

The lower sensitivity of *S. aureus* in mature biofilm, verified in our study, confirms the importance of the active microbial metabolism for VIO action, as the bacteria inside the biofilm have reduced metabolic activity [21]. The influence of cell metabolism on VIO activity is also confirmed by the VIO ability to inhibit biofilm formation.

Considering the occurrence of possible VIO decomposition after 8 h of incubation, as well as the possibility that VIO inhibitory action be favored by the high cell metabolism rate, one can infer that cell growth inhibition observed after 24 h, when cell division is minimal, may be associated with the high nutritional depletion and metabolite concentration.

VIO showed inhibitory activity in all stages of *S. aureus* growth. In these assays, the substance is added at different stages of growth and its activity is assessed after 4 and 24 h. The fact that VIO was added to each of the different stages of bacterial growth might have prevented its decomposition and favored its action, even at the low bacterial metabolism stages. Thus, an initial decrease in the microbial population (up to 4 h) was demonstrated for all phases. This was not observed when VIO was added only at the beginning of incubation, in the time–kill assay, which reinforces the idea that VIO may have degraded after 4–8 h, in microbial culture, under the experimental conditions.

The ability of *S. aureus* to form biofilms is a major virulence factor and contributes to the success of the infectious process [20]. The greater resistance of *S. aureus* in the mature biofilm matrix, when compared to that of its cells in planktonic culture, can be attributed to the ability of the bacterial biofilm to limit the access and dissemination of antimicrobial agents in its interior. In the biofilm, the cells are protected from antimicrobial agents, environmental conditions, and from the host’s immune response, and can display an increase of up to 1000 times the antimicrobial resistance rate [22].

In this study, we demonstrate that VIO has an excellent antimicrobial activity against *S. aureus* in intensive metabolism (exponential growth phase), possibly showing that its mechanism of action involves the synthesis of macromolecules. However, we also verified VIO antimicrobial action against *S. aureus* at all growth stages, although at a lower intensity, suggesting the existence of more than one antimicrobial mechanism of action for VIO.

## 4. Materials and Methods

### 4.1. Violacein

VIO extracted from the strain *Chromobacterium violaceum* ATCC 12472 was purified and characterized by nuclear magnetic resonance (NMR) [23].

### 4.2. Bacterial Strains

This study used four reference strains: *S. aureus* ATCC 6538P and *S. aureus* ATCC 14458 (oxacillin-susceptible strains-OSSA), *S. aureus* ATCC 33591 and *S. aureus* CCBH 5330 (oxacillin-resistant strains-ORSA).

### 4.3. Determination of Minimum Inhibitory Concentration (MIC) and Minimum Bactericidal Concentration (MBC)

The MIC was determined using the microdilution method in culture broth [24]. Aliquots of the microbial suspensions (10^6^ CFU/mL) in Brain–Heart Infusion Broth (BHI) (Merck Millipore Corporation) were added to the wells containing 95 µL of BHI and 100 µL of VIO (0.019–40 µg/mL), in 0.1% Dimethyl Sulfoxide (DMSO). The microplates were incubated for 24 h/37 °C. The MIC was considered as the lowest VIO concentration capable of completely inhibiting microbial growth. As experiment controls, sterile BHI broth, amikacin, and 0.1% DMSO were used.

The MBC was determined by counting viable cells [25]. Briefly, 5 μL of inoculum were collected and plated. The MBC was regarded as the lowest VIO concentration capable of determining the death of 99.9% of *S. aureus* cells of the initial inoculum. The assays were performed in triplicate.

### 4.4. Time–Kill Assay

Aliquots of 20 μL of VIO (MIC, 2× MIC, 4× MIC) were added to the microplate wells containing 100 µL of BHI broth and 80 µL of bacterial suspension (10^6^ CFU/mL). The microplates were incubated at 37 °C and 10 μL aliquots were removed, diluted in 0.85% sterile saline solution and plated for colony counting, at predetermined time intervals (0, 2, 4, 6, 8, 10, 12, and 24 h) [26]. Microbial cultures with no exposure to VIO were used as experimental control. The assays were performed in triplicate and the results were expressed in log_10_ CFU/mL.

### 4.5. Determination of VIO Effect on S. aureus at Different Stages of Microbial Metabolism

A microbial suspension was exposed to VIO MIC. VIO was added at the start of the incubation period (*t* = 0) and after 4, 8, and 24 h and microbial growth was determined after 4 and 24 h of exposure. Cultures of *S. aureus*, without VIO addition and submitted to the same experimental conditions were used as control [27]. The experiment was performed in triplicate and the results expressed as log_10_ CFU/mL.

### 4.6. Determination of Minimum Biofilm Inhibitory Concentration (MBIC) 

The effect of VIO on biofilm formation was assessed by determining the MBIC [28]. The biofilm was obtained and its mass quantified using the crystal violet technique [29]. The strains were cultivated in Tryptic Soy Broth (TSB) with 1% (*p*/*v*) of glucose for 24 h/37 °C. Aliquots of 100 μL of the culture and 100 μL of VIO (0.019 to 80 µg/mL) were added to microplates and incubated for 24 h/37 °C. The wells were washed and 200 μL of 99% methanol was added for 15 min. After, aliquots of 200 μL of crystal violet solution (CV) 2% (*v*/*v*) were added for 15 min. The CV uptake by the bacterial cells was released by adding 160 μL of 33% acetic acid and optical density reading was performed at 570 (OD570) on a microplate reader (Model Synergy HT, Biotek, Winooski, VT, USA). MBIC was regarded as the lowest concentration of VIO capable of inhibiting the formation of bacterial biofilm and the experiments were performed in triplicate.

### 4.7. Determination of Minimum Biofilm Eradication Concentration (MBEC)

MBEC was determined to evaluate VIO effect on mature biofilm viability [30]. The *S. aureus* ATCC 6538P strains were grown in TSB supplemented with 1% (*p*/*v*) of glucose (24 h/37 °C). Aliquots of 100 μL (10^6^ CFU/mL) were transferred into microplates and incubated for 72 h/37 °C. 50 µL of TSB and 50 µL of VIO (10–160 µg/mL) were added to the wells containing biofilm. The microplates were incubated for 24 h/37 °C and the viability of the remaining biofilms was measured by counting the colonies. Biofilms not treated with VIO were used as controls. MBEC was considered the lowest VIO concentration able to prevent microbial growth. The experiments were performed in triplicate and the results were expressed in log_10_ CFU/mL.

### 4.8. Biofilm Time–Kill Assay

VIO aliquots of 20 µL (0.5, 1, and 2× MBEC) were added to microplates containing 100 µL of TSB and mature biofilm of *S. aureus* ATCC 6538P, formed in 72 h as previously described. The microplates were incubated at 37 °C, and after 24 h the remaining biofilm viability was measured by counting the colonies at the time of VIO addition and at predetermined time intervals (30, 60, 90, 120, and 150 min) after VIO addition. Aliquots of the biofilm serial dilutions were plated and the counting of colonies was performed after 24 h/37 °C. Biofilms not treated with VIO were used as controls. The experiments were performed in triplicate and the results were expressed in log_10_ CFU/mL [30].

### 4.9. Statistical Analysis

Statistical analyses were performed using one-way ANOVA with post hoc Bonferroni test. The data were performed in triplicate. The charts are shown as mean ± standard deviation. Data were considered significant when *p* < 0.05.

## 5. Conclusions

VIO is a promising molecule for the development of an antimicrobial drug for the eradication of *S. aureus* biofilm infections. However, while its action is enhanced by the active cell metabolism, it is also present in cells with reduced metabolism.

## Figures and Tables

**Table 1:** ^1^H- and ^13^C-NMR spectrals data of VIO.

	HSQC		HMBC	
	**δ_C_**	**δ_H_ (Mult, *J*/Hz)**	**^2^*J*_CH_**	**^3^*J*_CH_**
**2**	129.7	8.06 (*d* 3.1)		
**3**	105.8	—	H-2	NH-1, H-13, H-5
**4**	125.6	—		NH-1, H-2, H-8
**5**	104.6	7.23 *d* (2.0)		OH, H-7
**6**	152.9	—	OH, H-5, H-7	H-8
**7**	113.1	6.79 *dd* (8.7, 2.0)		OH, H-5
**8**	113.4	7.35 *d* (8.7)		
**9**	131.6	—	NH-1	H-2, H-5, H-7
**11**	171.7	—	NH-10	H-13
**12**	137.0	—	H-13	NH-10
**13**	97.0	7.55 *d* (2.0)		NH-10
**14**	147.7	—	NH-10, H-13	
**16**	170.3	—	NH-15	
**17**	118.7	—		NH-15, H-19
**18**	122.4	—		NH-15, H-20, H-22
**19**	126.4	8.93 *d* (8.0)	H-20	H-21
**20**	120.9	6.95 *td* (8.0, 1.0)		H-22
**21**	129.4	7.20 *td* (8.0, 1.0)	H-20	H-19
**22**	109.1	6.83 *d* (8.0)		H-20
**23**	141.8	—	NH-15	H-19, H-21
**NH-1**		11.88 (*s*)		
**NH-10**		10.72 (*s*)		
**NH-15**		10.60 (*s*)		
**OH**		9.33 (*s*)		

DMSO-*d*_6_, 500 e 75 MHz.

**Table 2:** VIO antimicrobial activity on planktonic cultures and biofilms of *S. aureus*.

Microbial Strain	VIO μg/mL		
	MIC	MBC	MBIC
*S. aureus* ATCC 6538P (OSSA)	1.25	5	1.25
*S. aureus* ATCC 14458 (OSSA)	2.5	10	2.5
*S. aureus* ATCC 3359 (ORSA)	20	40	20
*S. aureus* CCBH 5330 (ORSA)	5	20	5

MIC: minimum inhibitory concentration; MBC: minimum bactericidal concentration; MBIC: minimum biofilm inhibitory concentration; ATCC: American type culture collection; CCBH: culture collection of hospital-acquired bacteria. OSSA: oxacillin-susceptible strain, ORSA: oxacillin-resistant strain. Assays were performed in triplicate.

## Supplementary Materials

The following are available online. ^1^H- and ^13^C-NMR of VIO.
