# Supplementary material for: Antimicrobial Effects of Violacein against Planktonic Cells and Biofilms of Staphylococcus aureus
Source: Molecules. 2017 Sep 25;22(10):1534. doi: 10.3390/molecules22101534 (PMC6151432; doi:10.3390/molecules22101534)
Supplement: Supplementary file 1 [file molecules-22-01534-s001.pdf]

Supplementary files

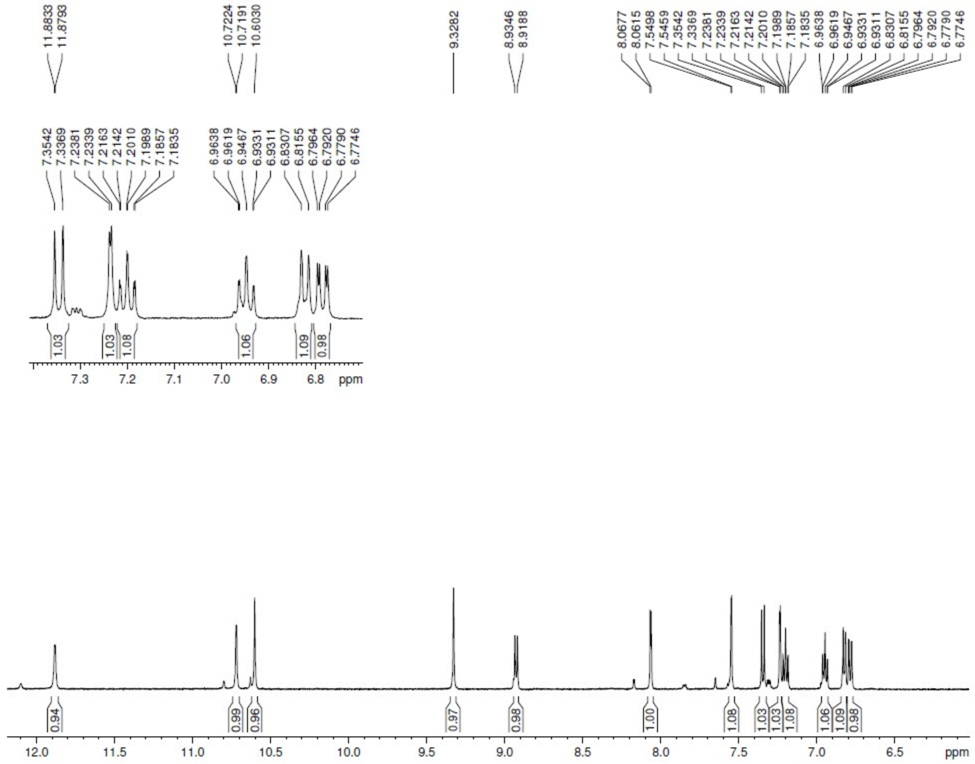

Figura 1. ^{1}H-NMR spectral of VIO (500 MHz, DMSO).

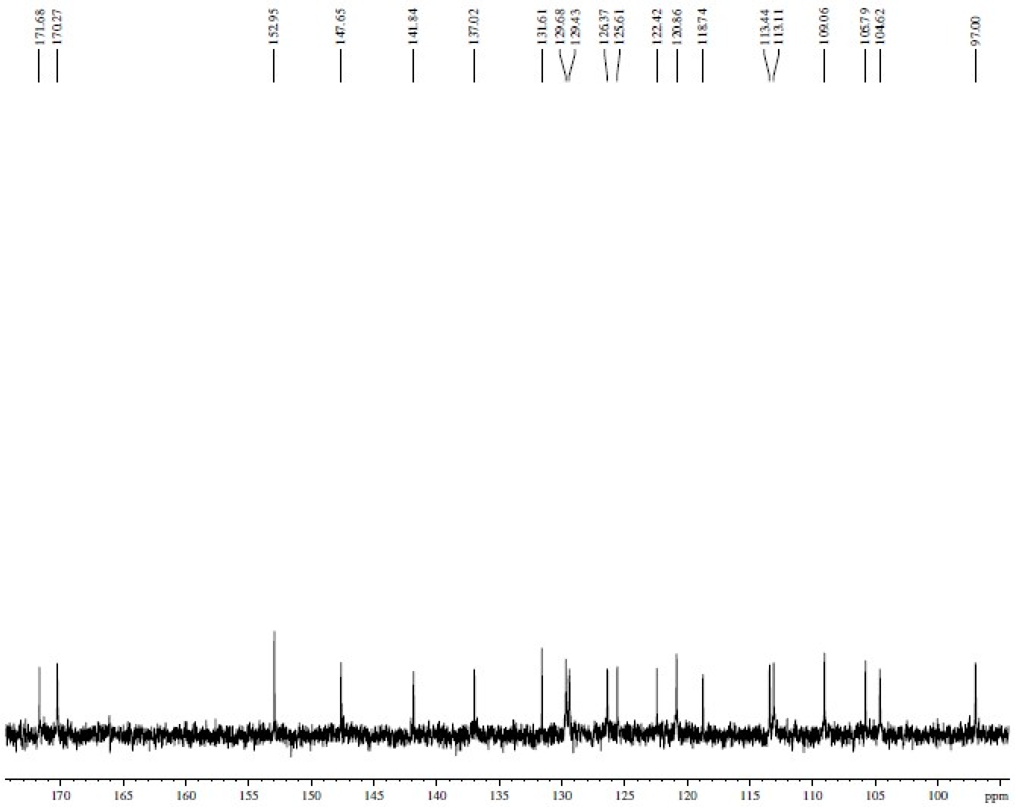

Figure 2. ^{13}C-NMR spectral of VIO (75 MHz, DMSO).
